# A Model for SARS-CoV-2 Infection with Treatment

**DOI:** 10.1155/2020/1352982

**Published:** 2020-09-01

**Authors:** Amar Nath Chatterjee, Fahad Al Basir

**Affiliations:** ^1^Department of Mathematics, K.L.S. College, Nawada, Magadh University, Bodh Gaya, Bihar 805110, India; ^2^Department of Mathematics, Asansol Girls' College, Asansol-4, West Bengal 713304, India

## Abstract

The current emergence of coronavirus (SARS-CoV-2) puts the world in threat. The structural research on the receptor recognition by SARS-CoV-2 has identified the key interactions between SARS-CoV-2 spike protein and its host (epithelial cell) receptor, also known as angiotensin-converting enzyme 2 (ACE2). It controls both the cross-species and human-to-human transmissions of SARS-CoV-2. In view of this, we propose and analyze a mathematical model for investigating the effect of CTL responses over the viral mutation to control the viral infection when a postinfection immunostimulant drug (pidotimod) is administered at regular intervals. Dynamics of the system with and without impulses have been analyzed using the basic reproduction number. This study shows that the proper dosing interval and drug dose both are important to eradicate the viral infection.

## 1. Introduction

A novel coronavirus named SARS-CoV-2 (an interim name proposed by WHO (World Health Organization)) became a pandemic since December 2019. The first infectious respiratory syndrome was recognized in Wuhan, Hubei province of China. Dedicated virologists identified and recognized the virus within a short time [1]. The SARS-CoV-2 is a single-stranded RNA virus genome which is closely related to severe acute respiratory syndrome- (SARS-) CoV [2]. The infection of SARS-CoV-2 is associated with a SARS-CoV-like a disease with a fatality rate of 3.4% [3]. The World Health Organization (WHO) have named the disease as COVID-19 and declared it as a public health emergency worldwide [4].

The common symptoms of COVID-19 are fever, fatigue, dry cough, and myalgia. Also, some patients suffer from headaches, abdominal pain, diarrhea, nausea, and vomiting. In the acute phase of infection, the disease may lead to respiratory failure which leads to death also. From clinical observation, within 1-2 days after patient symptoms, the patient becomes morbid after 4-6 days and the infection may clear within 18 days [5] depending on the immune system. Thus, appropriate quarantine measure for a minimum of two weeks is taken by the public health authorities for inhibiting community spread [6].

In [1], Zhou et al. identified the respiratory tract as the principal infection site for COVID-19 infection. SARS-CoV-2 infects primary human airway epithelial cells. The angiotensin-converting enzyme 2 (ACE2) receptor of epithelial cells plays an important role in cellular entry [1, 7]. It has been observed that ACE2 could be expressed in the oral cavity. ACE2 receptors are higher in the tongue than buccal and gingival tissues. These findings imply that the mucosa of the oral cavity may be a potentially high-risk route of COVID-19 infection. Thus, epithelial cells of the tongue are the major routes of entry for COVID-19. Zhou et al. [1] also reported that SARS-CoV-2 spikes (S) bind with the ACE2 receptor of epithelial cells with high affinity. The bonding between S (spikes) of SARS-CoV-2 and ACE2 [7] results from the fusion between the viral envelope and the target cell membrane, and the epithelial cells become infected. The S protein plays a major role in the induction of protective immunity during the infection of SARS-CoV-2 by eliciting neutralization antibody and T cell responses [8]. The S protein is not only capable of neutralizing antibody, but it also contains several immunogenic T cell epitopes. Some of the epitopes are found in either the S1 or S2 domain. These proteins are useful for SARS-CoV-2 drug development [9].

We know that virus clearance after acute infection is associated with strong antibody responses. Antibody responses have the potential to control the infection [10]. Also, CTL responses help to resolve infection and virus persistence caused by weak CTL responses [11]. Antibody responses against SARS-CoV-2 play an important role in preventing the viral entry process [8]. Hsueh et al. [2] found that antibodies block viral entry by binding to the S glycoprotein of SARS-CoV-2. To fight against the pathogen SARS-CoV-2, the body requires SARS-CoV-2-specific CD4^+^ T helper cells for developing this specific antibody [8]. Antibody-mediated immunity protection helps the anti-SARS-CoV serum to neutralize COVID-19 infection. Besides that, the role of T cell responses in COVID-19 infection is very much important. Cytotoxic T lymphocyte (CTL) responses are important for recognizing and killing infected cells, particularly in the lungs [8]. But the kinetics of the CTL responses and antibody responses during SARS-CoV-2 infection is yet to be explored. Our study will focus on the role of CTL and its possible implication on treatment and drug development. The drug that stimulates the CTL responses represents the best hope for control of COVID-19. Here, we have modeled the situation where CTLs can effectively control the viral infection when the postinfection drug is administered at regular intervals.

Mathematical modeling with real data can help in predicting the dynamics and control of an infectious disease [12, 13]. A four-dimensional dynamical model for a viral infection is proposed by Tang et al. [14] for MERS-CoV mediated by DPP4 receptors. In the case of SARS-CoV-2, the infection process is almost similar with MERS-CoV and SARS-CoV. For SARS-CoV-2 infection, the ACE2 receptors of epithelial cells are the major target area.

Since the dynamics of the disease transmission of SARS-CoV-2 in the cellular level is yet to be explored, we investigate the system in the light of the previous literature of [14–18] to formulate the dynamic model which plays a significant role in describing the interaction between uninfected cells, free virus, and CTL responses. We propose a novel deterministic model which describes the cell biological infection of SARS-CoV-2 with epithelial cells and the role of the ACE2 receptor.

We explained the dynamics in the acute infection stage. It has been observed that CTLs proliferate and differentiate antibody production after they encounter antigens. Here, we investigate the effect of CTL responses over the viral mutation to control viral infection when a postinfection drug is administered at regular intervals by a mathematical perspective.

It is clinically evident that immunostimulants play a crucial role in the case of respiratory disease. Among the currently available immunostimulants, pidotimod is the most effective for the respiratory disease [19]. Pidotimod increases the level of immunoglobulins (IgA, IgM, and IgG) and activates the CTL responses to fight against the disease.

In this article, we have considered the infection dynamics of SARS-CoV-2 infection in the acute stage. We have used impulsive differential equations to study the immunostimulant drug dynamics and the effects of perfect drug adherence. In recent years, the effects of perfect adherence have been studied by using impulsive differential equations in [20–26]. With the help of impulsive differential equations, the effect of maximal acceptable drug holidays and optimal dosage can be found more precisely [20, 26].

The article is organized as follows. The very next section contains the formulation of the impulsive mathematical model. Dynamics of the system without impulses has been provided in Section 3. The system with impulses has been analyzed in Section 4. Numerical simulations, on the basis of the outcomes of Sections 3 and 4, have been included in Section 5. Discussion in Section 6 concludes the paper.

## 2. Model Formulation

As discussed in the previous section, we propose a model considering the interaction between epithelial cells and SARS-CoV-2 virus along with lytic CTL responses over the infected cells. We consider five populations, namely, the uninfected epithelial cells *T*(*t*), infected cells *I*(*t*), ACE2 receptor of the epithelial cells *E*(*t*), SARS-CoV-2 virus V(t) and CTLs against the pathogen *C*(*t*).

In this model, we consider which represents the concentration of ACE2 on the surface of uninfected cells, which can be recognized by the surface spike (S) protein of SARS-CoV-2 [27].

It is assumed that the susceptible cells are produced at a rate *λ*_1_ from the precursor cells and die at a rate *d*_*T*_. The susceptible cells become infected at a rate *βE*(*t*)*V*(*t*)*T*(*t*). The constant *d*_*I*_ is the death rate of the infected cells. Infected cells are also cleared by the body's defensive CTLs at a rate *p*.

The infected cells produce new viruses at the rate *md*_*I*_ during their life, and *d*_*V*_ is the death rate of new virions, where *m* is any positive integer. It is also assumed that ACE2 is produced from the surface of uninfected cells at the constant rate *λ*_2_ and the ACE2 is destroyed, when free viruses try to infect uninfected cells, at the rate *θβE*(*t*)*V*(*t*)*T*(*t*) and is hydrolyzed at the rate *d*_*E*_*E*.

CTL proliferation in the presence of infected cells is described by the term

(1) $$\begin{array}{c} \alpha IC\left(1-\frac{C}{C_{\max}}\right), \end{array}$$

which shows the antigen-dependent proliferation. Here, we consider the logistic growth of CTL with *C*_max_ as the maximum concentration of CTL, and *d*_*c*_ is its rate of decay.

With the above assumptions, we have the following mathematical model characterizing the SARS-CoV-2 dynamics:

(2) $$\begin{array}{c} \frac{dT}{dt}=\lambda_{1}-\beta EVT-d_{T}T,\\ \frac{dI}{dt}=\beta EVT-d_{I}I-pIC,\\ \frac{dV}{dt}=md_{I}I-d_{V}V,\\ \frac{dE}{dt}=\lambda_{2}-\theta \beta EVT-d_{E}E,\\ \frac{dC}{dt}=\alpha IC\left(1-\frac{C}{C_{\max}}\right)-d_{c}C. \end{array}$$

A short description of the model parameters and their values is shown in Table 1. We now modify the above model by incorporating pulse periodic drug dosing using impulsive differential equations [28, 29].

We consider the perfect adherence behavior of the immunostimulant drug for SARS-CoV-2-infected patients at fixed drug dosing times *t*_*k*_, *k* ∈ *ℕ*.

We assume that CTL cells increase by a fixed amount *ω*, which is proportional to the total number of CTLs that the drug can stimulate. Thus, the above model takes the following form:

(3) $$\begin{array}{c} \frac{dT}{dt}=\lambda_{1}-\beta EVT-d_{T}T,\\ \frac{dI}{dt}=\beta EVT-d_{I}I-pIC,\\ \frac{dV}{dt}=md_{I}I-d_{V}V,\\ \frac{dE}{dt}=\lambda_{2}-\theta \beta EVT-d_{E}E,\\ \frac{dC}{dt}=\alpha IC\left(1-\frac{C}{C_{\max}}\right)-d_{c}C,\;t\neq t_{k},\\ C\left(t_{k}^{+}\right)=\omega +C\left(t_{k}^{-}\right),\;t=t_{k}. \end{array}$$

Here, *C*(*t*_*k*_^−^) denotes the CTL cell concentration immediately before the impulse, *C*(*t*_*k*_^+^) denotes the concentration after the impulse, and *ω* is the fixed amount which is proportional to the total number of CTLs the drug stimulates at each impulse time *t*_*k*_, *k* ∈ *ℕ*.

**Remark 1 .**

It can be noted that when there is no drug application in the system, model (3) becomes model (2).

## 3. Analysis of the System without the Drug

In this section, we analyze the dynamics of the system without impulses, i.e., system (1). We have derived the basic reproduction number for the system. Stability of equilibria is discussed using the number.

### 3.1. Existence of Equilibria

Model (2) has three steady states, namely, (i) the disease-free equilibrium *E*_1_(*λ*_1_/*d*_*T*_, 0, 0, *λ*_2_/*d*_*E*_, 0); (ii) with $\bar{E}>d_{T}d_{V}/\beta \lambda_{1}m$, there is a CTL response-free equilibrium, $E_{2}\left(\bar{T},\bar{I},\bar{V},\bar{E},0\right)$, where

(4) $$\begin{array}{c} \bar{T}=\frac{d_{V}}{\beta m\bar{E}},\\ \bar{I}=\frac{\beta \lambda_{1}m\bar{E}-d_{T}d_{V}}{\beta d_{I}m\bar{E}},\\ \bar{V}=\frac{\beta \lambda_{1}m\bar{E}-d_{T}d_{V}}{\beta d_{V}\bar{E}},\\ \bar{E}=\frac{-\left(\theta \beta \lambda_{1}m-\beta \lambda_{2}m\right)+\sqrt{{\left(\theta \beta \lambda_{1}m-\beta \lambda_{2}m\right)}^{2}+4\beta md_{T}d_{V}d_{E}\theta }}{2\beta d_{E}m}; \end{array}$$

and (iii) the endemic equilibrium *E*^∗^ which is given by

(5) $$\begin{array}{c} T^{*}=\frac{\lambda_{1}\alpha -d_{I}\alpha I^{*}-pd_{c}}{d_{T}\alpha }, \end{array}$$

(6) $$\begin{array}{c} C^{*}=\frac{\left(\alpha I^{*}-d_{c}\right)C_{\max}}{\alpha I^{*}}, \end{array}$$

where *I*^∗^ is the positive root of the cubic equation

(7) $$\begin{array}{c} L_{0}I^{3}+L_{1}I^{2}+L_{2}I+L_{3}=0, \end{array}$$

with

(8) $$\begin{array}{c} L_{0}=-\alpha^{2}\theta \beta d_{I}^{3}m,\\ L_{1}=-2\alpha \theta \beta d_{I}^{2}d_{c}mp+\alpha^{2}\theta \beta d_{I}^{2}\lambda_{1}m+\alpha^{2}\beta d_{I}^{2}\lambda_{2}m,\\ L_{2}=\alpha^{2}d_{T}d_{I}d_{V}d_{E}+\alpha \theta \beta d_{I}d_{c}\lambda_{1}mp+\alpha \beta d_{I}d_{c}\lambda_{2}mp-\alpha^{2}\beta d_{I}\lambda_{1}\lambda_{2}m-\theta \beta d_{I}d_{c}^{2}mp^{2},\\ L_{3}=\alpha d_{T}d_{V}d_{E}d_{c}p. \end{array}$$

**Remark 2 .**

Note that *L*_0_ < 0 and *L*_3_ > 0. Thus, equation (7) has at least one positive real root. If *L*_1_ > 0 and *L*_2_ < 0, then (3) can have two positive roots. For a feasible endemic equilibrium, we also need

(9) $$\begin{array}{c} \min\left\{\frac{\lambda_{1}\alpha -pd_{c}}{d_{I}\alpha },\frac{\lambda_{2}\alpha -\theta pd_{c}}{\theta \alpha d_{I}}\right\}>I^{*}>\frac{d_{c}}{\alpha }. \end{array}$$

### 3.2. Stability of Equilibria

In this section, the characteristic equation at any equilibrium is determined for the local stability of system (2). Linearizing system (2) at any equilibrium *E*(*T*, *I*, *V*, *E*, *C*) yields the characteristic equation

(10) $$\begin{array}{c} \Delta \left(\xi \right)=\;|\;\xi I_{n}-A\;|\;=0, \end{array}$$

where *I*_*n*_ is the identity matrix and **A** = [*a*_*ij*_] is the following 5 × 5 matrix given by

(11) $$\begin{array}{c} A=\left[\begin{array}{ccccc} -\beta EV-d_{T} & 0 & -\beta ET & -\beta VT & 0\\ \; & & & & \\ \beta EV & -d_{I}-pC & \beta ET & \beta VT & -pI\\ \; & & & & \\ 0 & d_{I}m & -d_{v} & 0 & 0\\ \; & & & & \\ -\theta \beta EV & 0 & -\theta \beta ET & -\theta \beta VT-d_{E} & 0\\ \; & & & & \\ 0 & \alpha C\left(1-\frac{C}{C_{\max}}\right) & 0 & 0 & a_{55} \end{array}\right], \end{array}$$

with *a*_55_ = *αI*(1 − 2*C*/*C*_max_) − *d*_*c*_. We finally get the characteristic equation as

(12) $$\begin{array}{c} \psi \left(\xi \right)=\xi^{5}+A_{1}\xi^{4}+A_{2}\xi^{3}+A_{3}\xi^{2}+A_{4}\xi +A_{5}=0. \end{array}$$

The coefficients *A*_*i*_, *i* = 1, 2, ⋯, 5, are given in the appendix.

Looking at stability of any equilibrium *E*, the Routh-Hurwitz criterion gives that all roots of this characteristic equation (12) have negative real parts, provided the following conditions hold

(13) $$\begin{array}{c} A_{5}>0,\\ A_{1}A_{2}-A_{3}>0,\\ A_{3}\left(A_{1}A_{2}-A_{3}\right)-A_{1}\left(A_{1}A_{4}-A_{5}\right)>0,\\ \left(A_{1}A_{2}-A_{3}\right)\left(A_{3}A_{4}-A_{2}A_{5}\right)-{\left(A_{1}A_{4}-A_{5}\right)}^{2}>0. \end{array}$$

Let us define the basic reproduction number as

(14) $$\begin{array}{c} R_{0}=\frac{m\beta \lambda_{1}\lambda_{2}}{d_{T}d_{E}d_{V}}. \end{array}$$

Then, using (5), we can derived the following result.

**Theorem 1 .**

Disease-free equilibrium *E*_1_(*λ*_1_/*d*_*T*_, 0, 0, *λ*_2_/*d*_*E*_, 0) of model (2) is stable for *R*_0_ < 1 and unstable for *R*_0_ > 1.

At *E*_2_, one eigenvalue is −*d*_*c*_ and the rest of the eigenvalues satisfy the following equation:

(15) $$\begin{array}{c} \xi^{4}+B_{1}\xi^{3}+B_{2}\xi^{2}+B_{3}\xi +B_{4}=0. \end{array}$$

The coefficients *B*_*i*_, *i* = 1, 2, ⋯, 5, are given in the appendix.

Using the Routh-Hurwitz criterion, we have the following theorem:

**Theorem 2 .**

The CTL-free equilibrium, $E_{2}\left(\bar{T},\bar{I},\bar{V},\bar{E},0\right)$, is asymptotically stable if and only if the following conditions are satisfied:

(16) $$\begin{array}{c} B_{1}>0,\\ B_{2}>0,\\ B_{3}>0,\\ B_{4}>0,\\ B_{1}B_{2}-B_{3}>0,\\ \left(B_{1}B_{2}-B_{3}\right)B_{3}-B_{1}^{2}B_{4}>0. \end{array}$$

Denoting *A*_*i*_^∗^ = *A*_*i*_(*E*^∗^) and using (5), we have the following theorem establishing the stability of coexisting equilibrium *E*^∗^.

**Theorem 3 .**

The coexisting equilibrium *E*^∗^ is asymptotically stable if and only if the following conditions are satisfied:

(17) $$\begin{array}{c} A_{5}^{*}>0,\\ A_{1}^{*}A_{2}^{*}-A_{3}^{*}>0,\\ A_{3}^{*}\left(A_{1}^{*}A_{2}^{*}-A_{3}^{*}\right)-A_{1}^{*}\left(A_{1}^{*}A_{4}^{*}-A_{5}^{*}\right)>0,\\ \left(A_{1}^{*}A_{2}^{*}-A_{3}^{*}\right)\left(A_{3}^{*}A_{4}^{*}-A_{2}^{*}A_{5}^{*}\right)-{\left(A_{1}^{*}A_{4}^{*}-A_{5}^{*}\right)}^{2}>0. \end{array}$$

## 4. Dynamics of the System with Impulsive Drug Dosing

In this section, we consider the model system (3). Before analyzing the system, we first discuss the one-dimensional impulse system as follows:

(18) $$\begin{array}{c} \frac{dC}{dt}=\alpha IC\left(1-\frac{C}{C_{\max}}\right)-d_{c}C,\;t\neq t_{k},\\ C\left(t_{k}^{+}\right)=\omega +C\left(t_{k}^{-}\right),\;t=t_{k}. \end{array}$$

*C*(*t*_*k*_^−^) denotes the CTL responses immediately before the impulse drug dosing, *C*(*t*_*k*_^+^) denotes the concentration after the impulse, and *ω* is the dose that is taken at each impulse time *t*_*k*_, *k* ∈ *ℕ*.

We now consider the following linear system:

(19) $$\begin{array}{c} \begin{array}{cc} \frac{dC}{dt}=-d_{c}C, & t\neq t_{k} \end{array},\\ \begin{array}{cc} \Delta C=\omega , & t=t_{k} \end{array}, \end{array}$$

where Δ = *C*(*t*_*k*_^+^) − *C*(*t*_*k*_^−^). Let *τ* = *t*_*k*+1_ − *t*_*k*_ be the period of the campaign. The solution of system (10) is

(20) $$\begin{array}{c} C\left(t\right)=C\left(t_{k}^{+}\right)e^{-d_{c}\left(t-t_{k}\right)},\;\text{for }t_{k}<t\leq t_{k+1}. \end{array}$$

In presence of impulsive dosing, we can get the recursion relation at the moments of impulse as

(21) $$\begin{array}{c} C\left(t_{k}^{+}\right)=C\left(t_{k}^{-}\right)+\omega . \end{array}$$

Thus, the amount of CTL before and after the impulse is obtained as

(22) $$\begin{array}{c} C\left(t_{k}^{+}\right)=\frac{\omega \left(1-e^{-k\tau d_{c}}\right)}{1-e^{-\tau d_{c}}},\\ C\left(t_{k+1}^{-}\right)=\frac{\omega \left(1-e^{-k\tau d_{c}}\right)e^{-\tau d_{c}}}{1-e^{-\tau d_{c}}}. \end{array}$$

Thus, the limiting case of the CTL amount before and after one cycle is as follows:

(23) $$\begin{array}{c} \underset{k\to \infty }{\lim}C\left(t_{k}^{+}\right)=\frac{\omega }{1-e^{-\tau d_{c}}},\\ \underset{k\to \infty }{\lim}C\left(t_{k+1}^{-}\right)=\frac{\omega e^{-\tau d_{c}}}{1-e^{-\tau d_{c}}},\\ C\left(t_{k+1}^{+}\right)=\frac{\omega e^{-\tau d_{c}}}{1-e^{-\tau d_{c}}}+\omega =\frac{\omega }{1-e^{-\tau d_{c}}}. \end{array}$$

**Definition 1 .**

Let *Λ* ≡ (*S*_*u*_, *S*_*a*_, *I*, *C*) and *B*_0_ = [*B* : *R*_+_^4^ → *R*_+_]; then, we say that *B* belong to class *B*_0_ if the following conditions hold:

- *B* is continuous on (*t*_*k*_, *t*_*k*+1_] × *R*_+_^3^, *n* ∈ *N*, and for all *Λ* ∈ *R*^4^, lim_(*t*, *μ*)→(*t*_*k*_^+^, *Λ*)_*B*(*t*, *μ*) = *B*(*t*_*k*_^+^, *Λ*) exists
- *B* is locally Lipschitzian in *Λ*

We now recall some results for our analysis from [28, 29].

**Lemma 1 .**

Let *Z*(*t*) be a solution of system (9) with *Z*(0^+^) ≥ 0. Then, *Z*_*i*_(*t*) ≥ 0, *i* = 1, ⋯, 4, for all *t* ≥ 0. Moreover, *Z*_*i*_(*t*) > 0, *i* = 1, ⋯, 4, for all *t* > 0 if *Z*_*i*_(0^+^) > 0, *i* = 1, ⋯, 4.

**Lemma 2 .**

There exists a constant *γ* such that *T*(*t*) ≤ *γ*, *I*(*t*) ≤ *γ*, *V*(*t*) ≤ *γ* *E*(*t*) ≤ *γ*, and *C*(*t*) ≤ *γ* for each and every solution *Z*(*t*) of system (9) for all sufficiently large *t*.

**Lemma 3 .**

Let *B* ∈ *B*_0_ and also consider that

(24) $$\begin{array}{c} D^{+}B\left(t,Z\right)\leq j\left(t,B\left(t,Z\left(t\right)\right)\right),\;t\neq t_{k},\\ B\left(t,Z\left(t^{+}\right)\right)\leq \Phi_{n}\left(B\left(t,Z\left(t\right)\right)\right),\;t=t_{k}, \end{array}$$

where *j* : **R**_+_ × **R**_+_ → **R** is continuous in (*t*_*k*_, *t*_*k*+1_] for *e* ∈ **R**_+_^2^, *n* ∈ *N*, the limit lim_(*t*, *V*)→(*t*_*k*_^+^)_*j*(*t*, *g*) = *j*(*t*_*k*_^+^, *x*) exists, and *Φ*_*n*_^*i*^(*i* = 1, 2): **R**_+_ → **R**_+_ is nondecreasing. Let *y*(*t*) be a maximal solution of the following impulsive differential equation:

(25) $$\begin{array}{c} \frac{dx\left(t\right)}{dt}=j\left(t,x\left(t\right)\right),\;t\neq t_{k},\\ x\left(t^{+}\right)=\Phi_{n}\left(x\left(t\right)\right),\;t=t_{k},x\left(0^{+}\right)=x_{0}, \end{array}$$

existing on (0^+^, ∞). Then, *B*(0^+^, *Z*_0_) ≤ *x*_0_ implies that *B*(*t*, *Z*(*t*)) ≤ *y*(*t*), *t* ≥ 0, for any solution *Z*(*t*) of system (9). If *j* satisfies additional smoothness conditions to ensure the existence and uniqueness of solutions for (12), then *y*(*t*) is the unique solution of (12).

We now consider the following subsystem:

(26) $$\begin{array}{c} \frac{dC\left(t\right)}{dt}=-d_{c}C,\;t\neq t_{k},C\left(t_{k}^{+}\right)=C\left(t_{k}\right)+\omega ,C\left(0^{+}\right)=C_{0}. \end{array}$$

The lemma provided above gives the following result.

**Lemma 4 .**

System (13) has a unique positive periodic solution $\tilde{C}\left(t\right)$ with period *τ* and given by

(27) $$\begin{array}{c} \tilde{C}\left(t\right)=\frac{\omega \;\exp\;\left(-d_{c}\left(t-t_{k}\right)\right)}{1-\exp\;\left(-\tau d_{c}\right)},\;t_{k}<t\leq t_{k+1},\tilde{C}\left(0^{+}\right)=\frac{d_{c}}{1-\exp\;\left(-\tau d_{c}\right)}. \end{array}$$

We use this result to derive the following theorem.

**Theorem 4 .**

The disease-free periodic orbit $\left(\tilde{T},0,0,\tilde{E},\tilde{C}\right)$ of system (2) is locally asymptotically stable if

(28) $$\begin{array}{c} {\tilde{R}}_{0}<1, \end{array}$$

where

(29) $$\begin{array}{c} {\tilde{R}}_{0}=\frac{md_{I}\beta }{d_{T}d_{E}d_{V}\tau }\int_{0}^{\tau }\;\frac{\tilde{T}\tilde{E}}{d_{I}+p\tilde{C}}dt. \end{array}$$

**Proof**

Let the solution of system (9) without infected people be denoted by $\left(\tilde{T},0,0,\tilde{E},\tilde{C}\right)$, where

(30) $$\begin{array}{c} \tilde{C}\left(t\right)=\frac{\omega \exp\left(-d_{c}\left(t-t_{k}\right)\right)}{1-\exp\left(-\tau d_{c}\right)},\;t_{k}<t\leq t_{k+1}, \end{array}$$

with initial condition *C*(0^+^) as in Lemma 10. We now test the stability of the equilibria. The variational matrix at $\left(\tilde{T},0,0,\tilde{E},\tilde{C}\right)$ is given by

(31) $$\begin{array}{c} M\left(t\right)=\left[m_{ij}\right]=\left(\begin{array}{ccccc} -d_{T} & 0 & m_{13} & 0 & 0\\ \; & & & & \\ 0 & -\left(d_{I}+p\tilde{C}\right) & \beta \tilde{E}\tilde{T} & 0 & 0\\ \; & & & & \\ 0 & md_{I} & -d_{v} & 0 & 0\\ \; & & & & \\ 0 & 0 & m_{43} & -d_{E} & 0\\ \; & & & & \\ 0 & m_{52} & 0 & 0 & -d_{c} \end{array}\right). \end{array}$$

The monodromy matrix *ℙ* of the variational matrix *M*(*t*) is

(32) $$\begin{array}{c} \mathbb{P}\left(\tau \right)=I_{n}\exp\left(\int_{0}^{\tau }\;M\left(t\right)dt\right), \end{array}$$

where *I*_*n*_ is the identity matrix. Note that *m*_13_, *m*_43_, and *m*_52_ are not required for this analysis; therefore, we have not mentioned their expressions.

We can write *ℙ*(*τ*) = diag(*σ*_1_, *σ*_2_, *σ*_3_, *σ*_4_, *σ*_5_), where *σ*_*i*_, *i* = 1, 2, 3, 4, 5, are the Floquet multipliers and they are determined as

(33) $$\begin{array}{c} \sigma_{1}=\exp\left(-d_{T}\tau \right),\\ \sigma_{2,3}=\exp\left(\int_{0}^{\tau }\;\frac{1}{2}\left[-A\pm \sqrt{A^{2}-4B}\right]dt\right),\\ \sigma_{4}=\exp\left(-d_{E}\tau \right),\\ \sigma_{5}=\exp\left(-d_{c}\tau \right). \end{array}$$

Here, $A=d_{I}+d_{V}+p\tilde{C}$ and $B=d_{V}\left(d_{I}+p\tilde{C}\right)-md_{I}\beta \tilde{E}\tilde{T}$. Clearly, *λ*_1,4,5_ < 1. It is easy to check that *A*^2^ − 4*B* > 0, and if *B* ≥ 0 and hold, then we have *λ*_2,3_ < 1. Thus, according to Floquet theory, the periodic solution $\left(\tilde{T},0,0,\tilde{E},\tilde{C}\right)$ of system (9) is locally asymptotically stable if the conditions given in (14) hold.

## 5. Numerical Results and Discussion

**Figure 1:**
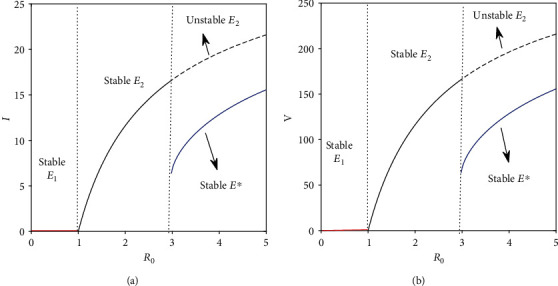
Existence and stability of equilibria is shown with respect to *R*_0_. Parameter values used in this figure are taken from Table 1 and *m* = 10. We have varied the value of *β* in (0.00001,0.0001).

**Figure 2:**
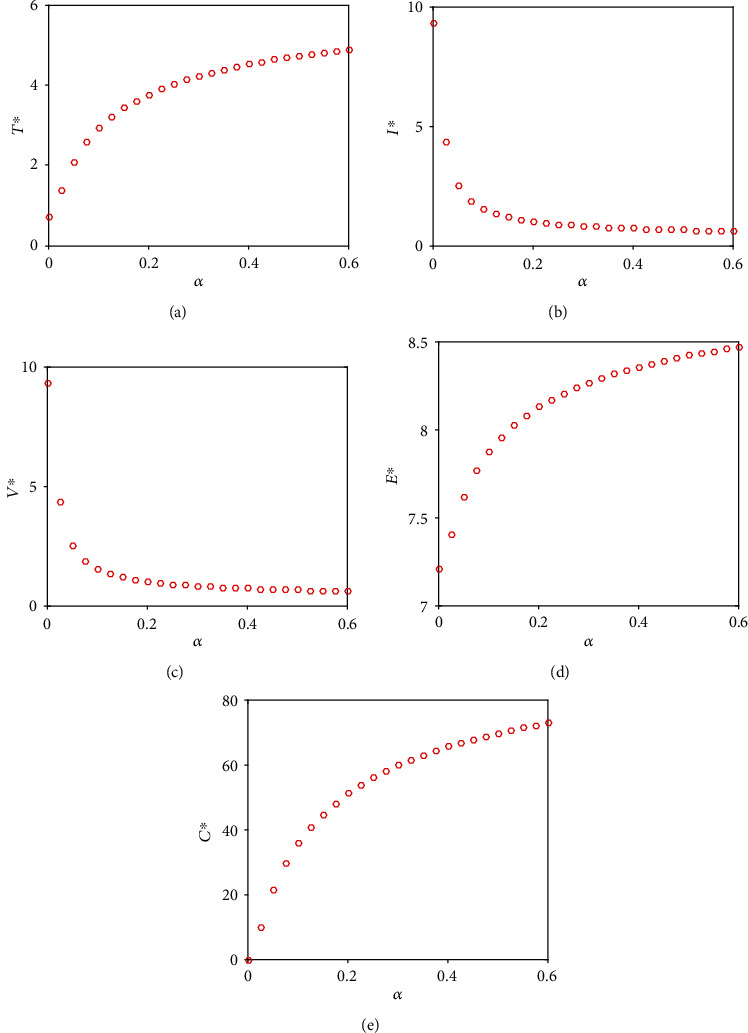
In the absence of the drug, the effect of the growth rate of CTL, i.e., *α* on the steady-state values of model population, is shown. Parameter values used in this figure are the same as Figure 1 except *α*.

**Figure 3:**
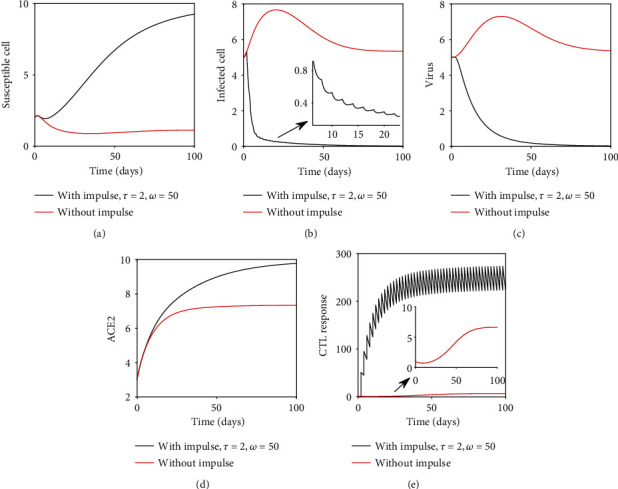
Numerical solution of the model system with and without the drug dose is shown taking parameters as in Figure 1. In this figure, *τ* = 2 and *ω* = 50.

**Figure 4:**
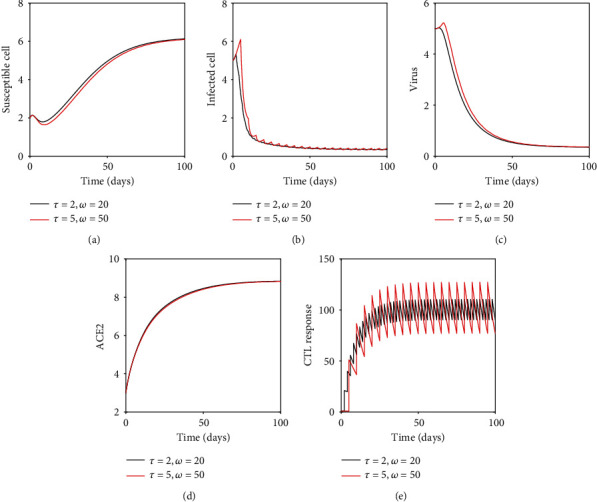
Numerical solution of the model system for different rates of drug dosing and different intervals of impulses.

In this section, we have observed the dynamical behaviors of the system without the drug (Figures 1 and 2) and with impulsive effect of the drug dose (Figures 3 and 4) through numerical simulations taking the parameters mainly from [14, 19, 30].

We have mainly focused on the role of CTL and its possible implication on the treatment and drug development. The drug that stimulates the CTL responses represents the best hope for control of COVID-19. Here, we have determined the situation where CTLs can effectively control the viral infection when the postinfection drug is administered at regular intervals.

Existence of equilibria of the system without the drug dose is shown for different values of basic reproduction number *R*_0_. In plotting Figure 1, we have varied the value of infection rate *β*. It is observed that for the lower infection rate (that corresponds to *R*_0_ < 1), disease-free equilibrium *E*_1_ is stable (corroborated with Theorem 3). It becomes unstable and ensures the existence of the CTL-free equilibrium *E*_2_ which is stable if *R*_0_ < 2.957 (which corresponds to *β* = 0.00005963) and unstable otherwise. (This satisfies Theorem 4.) Again, we see that when *E*_2_ is unstable, *E*^∗^ is feasible. Also, whenever *E*^∗^ exists, it is stable which verified the Theorem 5.

The effect of the immune response rate *α* is plotted in Figure 2. We observe that in the absence of the drug, the CTL count and ACE2 increase with increasing value of *α*. The steady-state value of infected cell *I*^∗^ and virus *V*^∗^ decreases significantly as *α* increases.

Due to the impulsive nature of the drugs, there are no equilibria of the system; i.e., population does not reach towards the equilibrium point, rather approach a periodic orbit. Hence, we evaluate equilibrium-like periodic orbits. There are two periodic orbits of system (3), namely, the disease-free periodic orbit and endemic periodic orbit. Here, our aim is to find the stability of the disease-free periodic orbit.

Figure 3 compares the system without and with impulse drug effect. In the absence of the drug, we observe that the CTL count approaches a stable equilibrium. Under regular drug dosing, the CTL count oscillates in an impulsive periodic orbit. Assuming perfect adherence, if the drug is sufficiently strong, both infected cell and virus population approach towards extinction. In this case, the total number of uninfected cells reaches its maximum level which implies that the system approaches towards its infection-free state (Theorem 11).

If we take sufficiently large impulsive interval *τ* = 5 days (keeping rate *ω* = 50 fixed, as in Figure 3) or lower dosage effect *ω* = 20 (keeping interval *τ* = 2 fixed, as in Figure 3), in both the cases, infection remains present in the system. Thus, the proper dosage of drug and optimal dosing interval are important for infection management.

## 6. Conclusion

In this article, the role of the immunostimulant drug (mainly pidotimod) during interactions between SARS-CoV-2 spike protein and epithelial cell receptor ACE2 in COVID-19 infection has been studied as a possible drug dosing policy. To reactivate the CTL responses during the acute infection period, immune activator drugs are delivered to the host system in an impulsive mode.

When the immunostimulant drug is administered, the best possible CTL responses can act against the infected or virus-producing cells to neutralize infection. This particular situation can keep the infected cell population at a very low level. In the proposed mathematical model, we have analyzed the optimal dosing regimen for which infection can be controlled.

From this study, it has been observed that when the basic reproduction ratio lies below one, we expect the system to attain its disease-free state. However, the system switches from the disease-free state to the CTL-free equilibrium state when 1 < *R*_0_ < 2.957. If *R*_0_ > 2.957, the CTL-free equilibrium moves to an endemic state (Figure 1).

Here, we have explored the immunostimulant drug dynamics by the help of impulsive differential equations. With the help of impulsive differential equations, we have studied how the effect of the maximal acceptable optimal dosage can be found more precisely. The impulsive system shows that the proper dosage and dosing intervals are important for the eradication of the infected cells and virus population which results in the control of the pandemic (Figure 3).

It has also been observed that the length of the dosing interval and the drug dose play a very decisive role to control and eradicate the infection. The most interesting prediction of this model is that effective therapy can often be achieved, even for low adherence, if the dosing regimen is adjusted appropriately (Figure 4). Also, if the treatment regimen is not adjusted properly, the therapy is not effective at all. This approach might also be applicable to a combination of antiviral therapy.

Future extension work of the combination of drug therapy should also include more realistic patterns of nonadherence (random drug holidays, imperfect timing of successive doses) and more accurate intracellular pharmacokinetics which leads towards better estimates of drug dosage and drug dosing intervals.

We end the paper with the quotation: “*This outbreak is a test of political*, *financial and scientific solidarity for the world to fight a common enemy that does not respect borders*..., *what matters now is stopping the outbreak and saving lives*,” by Dr. Tedros, Director General, WHO [31].

## Figures and Tables

**Table 1:** Set of parameter values used of numerical simulations.

Parameter	Explanation	Assigned value
*λ*_1_	Production rate of uninfected cell	5
*λ*_2_	Production rate of ACE2	1
*β*	Disease transmission rate	0.0001
*θ*	Bonding rate of ACE2	0.3
*d*_*T*_	Death rate of uninfected cells	0.1
*d*_*I*_	Death rate of infected cells	0.1
*d*_*V*_	Removal rate of virus	0.1
*d*_*E*_	Hydrolyzing rate of epithelial cells	0.1
*d*_*c*_	Decay rate of CTL	0.1
*p*	Killing rate of infected cells by CTL	0.01
*m*	Number of new virions produced	10-100
*α*	Proliferation rate of CTL	0.22
*C*_max_	Maximum proliferation of CTL	100

## Data Availability

The data used for supporting the findings are included within the article.

## Appendix

### Analysis of the System without the Drug

(A.1) $$\begin{array}{c} A_{1}=-\left(a_{11}+a_{22}+a_{33}+a_{44}+a_{55}\right),\\ A_{2}=a_{11}\left(a_{22}+a_{33}\right)+a_{23}a_{32}+a_{22}a_{33}-a_{14}a_{41}+\left(a_{11}+a_{22}+a_{33}\right)a_{44}-a_{25}a_{52}+\left(a_{11}+a_{22}+a_{33}+a_{44}\right)a_{55},\\ A_{3}=a_{32}\left(a_{11}a_{23}-a_{13}a_{21}-a_{24}a_{43}\right)-a_{11}a_{22}\left(a_{44}+a_{33}\right)+a_{14}a_{33}a_{41}+a_{44}\left(a_{23}a_{32}-a_{11}a_{33}-a_{22}a_{33}\right)+a_{25}a_{52}\left(a_{33}+a_{44}+a_{11}\right)-a_{11}a_{55}\left(a_{22}+a_{33}\right)+a_{23}a_{32}a_{55}-a_{22}a_{33}a_{55}-a_{44}a_{55}\left(a_{11}+a_{14}a_{41}a_{55}+a_{22}+a_{33}\right)+a_{14}a_{22}a_{41},\\ A_{4}=a_{32}a_{41}\left(a_{23}a_{14}-a_{13}a_{24}\right)-a_{22}a_{33}\left(a_{14}a_{41}-a_{44}a_{55}\right)-a_{14}a_{21}a_{32}a_{43}+a_{13}a_{21}a_{32}a_{44}-a_{11}\left(a_{23}a_{32}a_{44}+a_{25}a_{33}a_{52}\right)+a_{52}\left(a_{14}a_{25}a_{41}-a_{11}a_{25}a_{44}-a_{25}a_{33}a_{44}\right)+a_{13}a_{21}a_{32}a_{55}-a_{11}a_{55}\left(a_{23}a_{32}-a_{22}a_{33}\right)-a_{41}a_{55}\left(a_{14}a_{22}+a_{14}a_{33}\right)+a_{55}\left(a_{24}a_{32}a_{43}+a_{11}a_{22}a_{44}-a_{23}a_{32}a_{44}\right)+a_{11}a_{33}\left(a_{22}a_{44}+a_{44}a_{55}\right)+a_{11}a_{24}a_{32}a_{43},\\ A_{5}=a_{25}a_{33}\left(a_{11}a_{44}a_{52}-a_{14}a_{41}a_{52}\right)+\left(a_{14}a_{21}a_{32}-a_{11}a_{24}a_{32}\right)a_{43}a_{55}+a_{41}a_{55}\left(a_{13}a_{24}a_{32}-a_{14}a_{23}a_{32}+a_{14}a_{22}a_{33}\right)+a_{44}a_{55}\left(a_{11}a_{23}a_{32}-a_{13}a_{21}a_{32}-a_{11}a_{22}a_{33}\right). \end{array}$$

(A.2) $$\begin{array}{c} B_{1}=-\left(b_{11}+b_{22}+b_{33}+b_{44}\right),\\ B_{2}=b_{11}b_{22}-b_{23}b_{32}+b_{33}\left(b_{11}+b_{22}\right)-b_{14}b_{41}+b_{44}\left(b_{11}+b_{22}+b_{33}\right),\\ B_{3}=b_{32}\left(b_{11}b_{23}-b_{13}b_{21}\right)-b_{22}\left(b_{11}b_{33}-b_{14}b_{41}\right)+b_{14}b_{33}b_{41}-b_{24}b_{32}b_{43}+\left(b_{23}b_{32}-b_{11}b_{22}\right)b_{44}-b_{33}b_{44}\left(b_{11}+b_{22}\right),\\ B_{4}=b_{14}b_{41}\left(b_{23}b_{32}-b_{22}b_{33}\right)-b_{13}b_{24}b_{32}b_{41}-b_{14}b_{21}b_{32}b_{43}+b_{32}\left(b_{11}b_{24}b_{43}+b_{13}b_{21}b_{44}\right)-b_{44}b_{11}\left(b_{23}b_{32}-b_{22}b_{33}\right). \end{array}$$
